# Supplementary material for: Activation mechanism of a small prototypic Rec-GGDEF diguanylate cyclase
Source: Nat Commun. 2021 Apr 12;12:2162. doi: 10.1038/s41467-021-22492-7 (PMC8041772; doi:10.1038/s41467-021-22492-7)
Supplement: Supplementary file 1 — Supplementary Information [file 41467_2021_22492_MOESM1_ESM.pdf]

Supplementary Figures

Supplementary Fig. 1

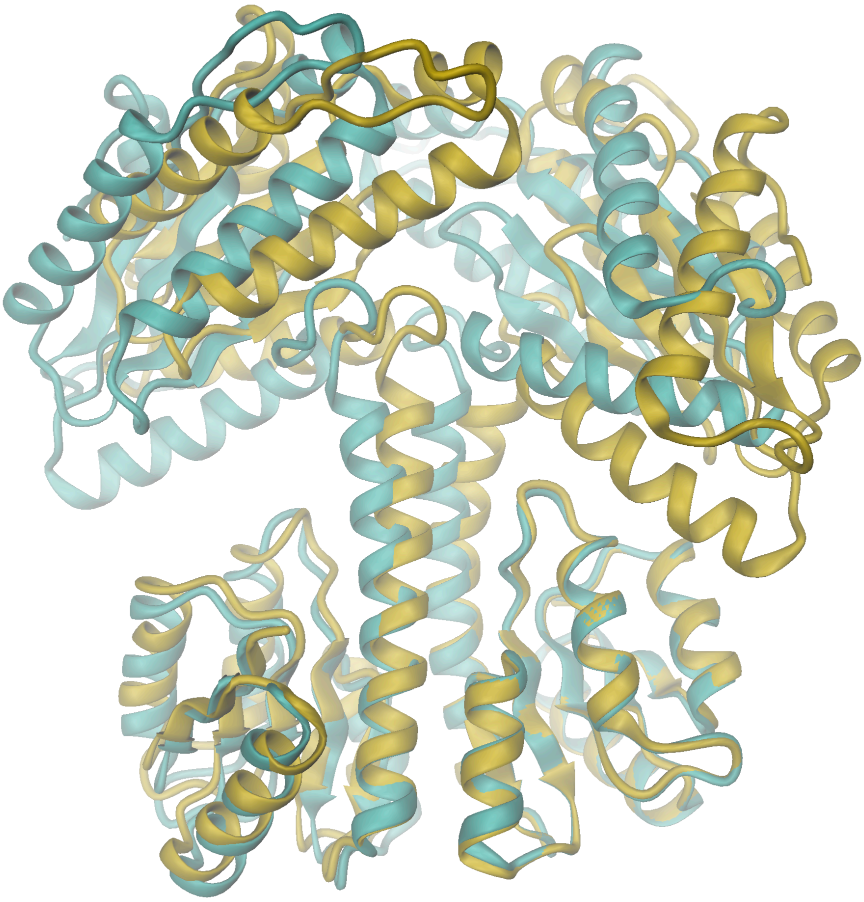

Supplementary Figure 1. Superimposition of the two dimers of the asymmetric unit of the DgcR'* crystal structure. Dimers AB and CD were superimposed on their Rec part and are represented in turquoise and gold, respectively.

Supplementary Fig. 2

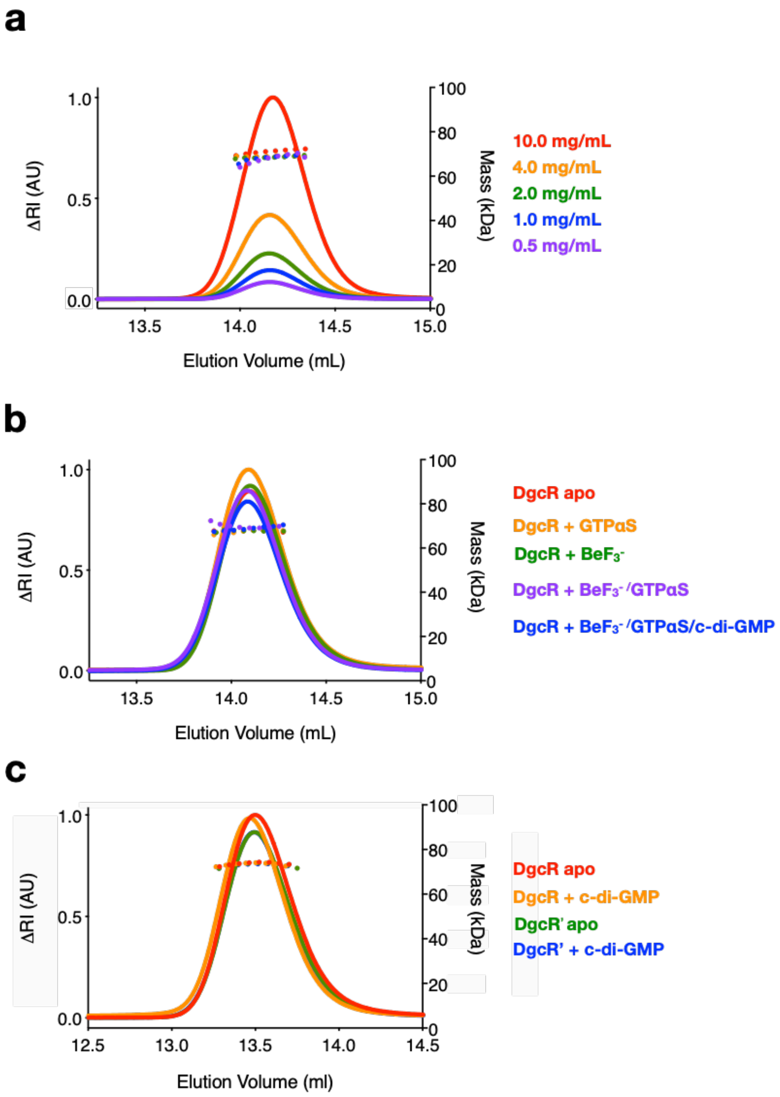

Supplementary Fig. 2. DgcR is a constitutive dimer. Oligomeric state of DgcR and DgcR' analyzed by SEC-MALS in 20 mM Tris pH 8.0, 5 mM $MgCl_2$,1mM DTT, and modified by BeF3- (see Material and Methods) where indicated. Molecular mass values (right axis) are represented by dotted lines and change in refractive index (left axis) by solid lines. **a)** DgcR measured using the indicated monomeric loading concentrations (corresponding to 0.274 mM to 0.014 mM) and an NaCl concentration of 500 mM. **b)** DgcR measured with 0.5 mg/ml loading concentration, 500 mM NaCl, and the indicated additional compounds. **c)** DgcR and DgcR' measured as in **b**), but a low salt concentration (20 mM NaCl). The elution volume is different to that in panels **a)** and **b)** due to a different instrument set up. But note, that the determined mass is the same in all experiments.

Supplementary Fig. 3

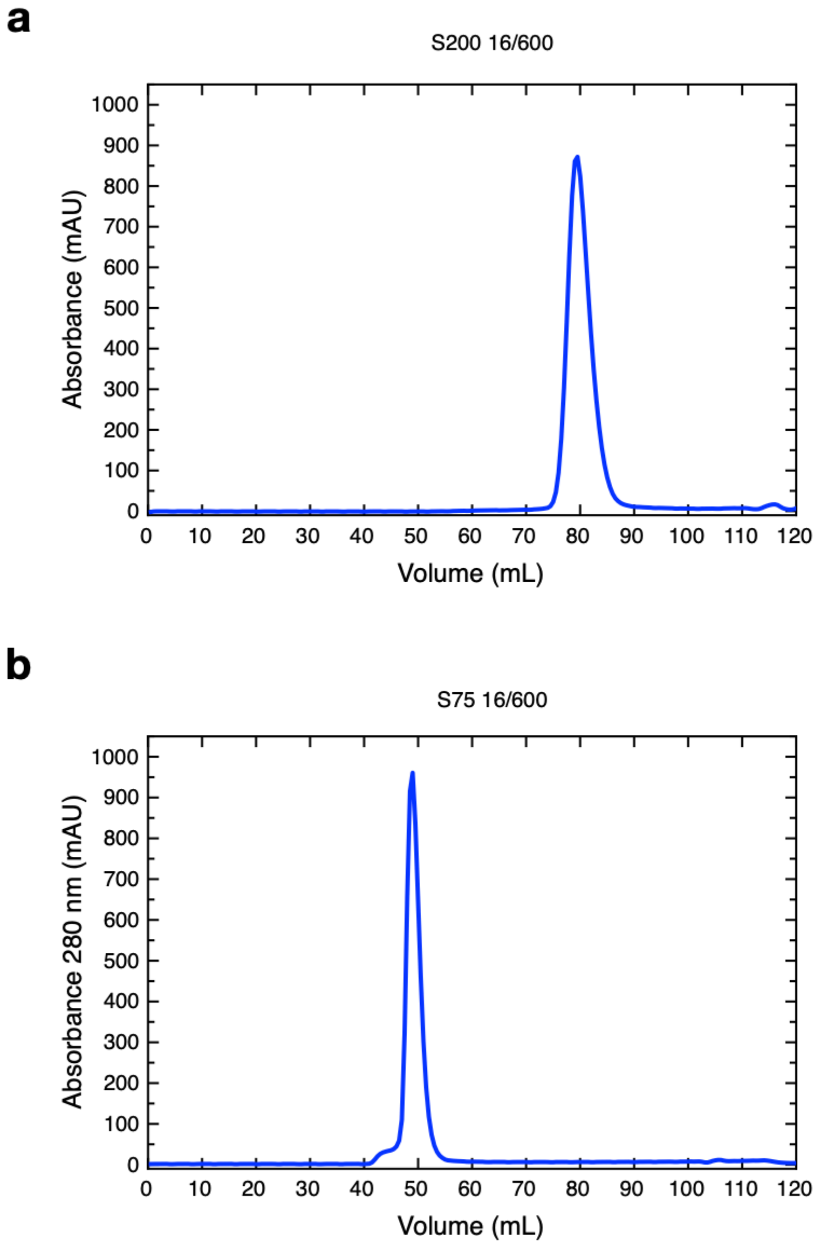

Supplementary Fig. 3. DgcR size exclusion purification. a) Purification in S200 16/60 (Cytiva Life Sciences) **b)** Purification in S75 16/60 (Cytiva Life Sciences).

Supplementary Fig. 4

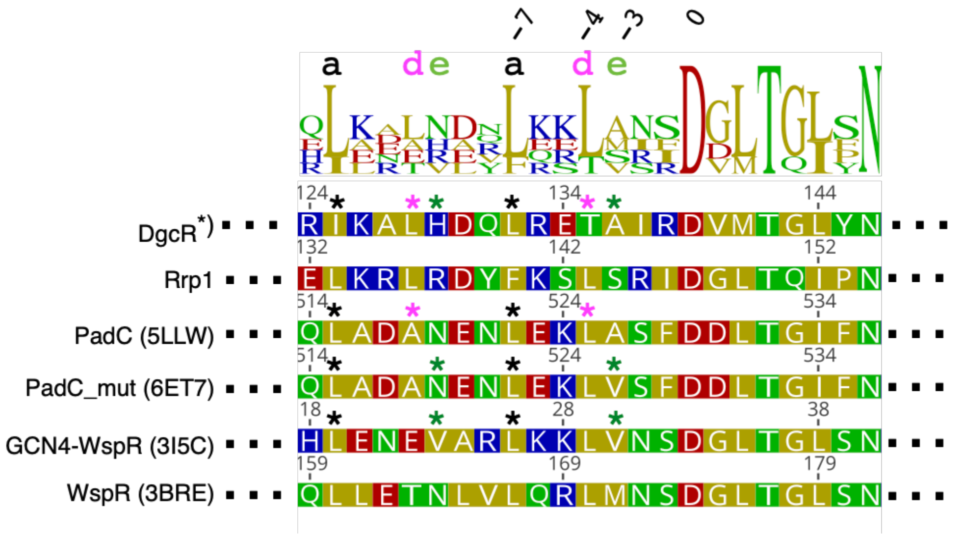

Supplementary Fig. 4. Alignment of selected DGCs comprising C-terminal end of Rec α5 and beginning of GGDEF domain. DgcR and 3 other well studied DGCs were included in the alignment: Rrp1 from *Borrelia burgdorferi*, PadC from *Idiomarina sp. A28L* and WspR from *Pseudomonas aeruginosa* (wild type and GCN4 hybrid). Sequence numbers relative to DxLT motif are given on the top. Asterisks denote crystallographically observed coiled-coil contacts (black: persistent contacts; colored: conditional contacts, i.e. contacts formed only in the native (pink) or activated (green) register).

) DgcR' and DgcR' of this study.

Supplementary Fig. 5

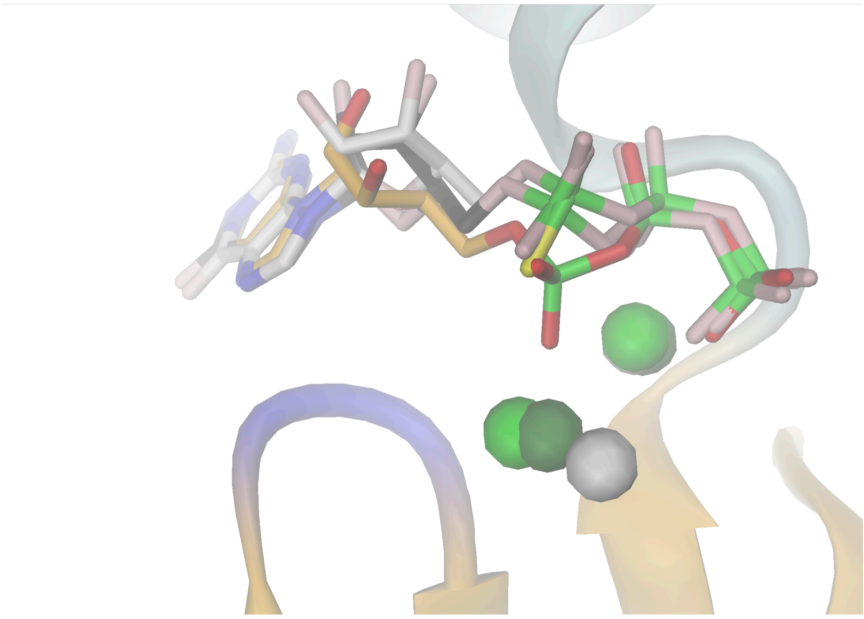

Supplementary Fig. 5. Superposition of GTP analog structures as bound to GGDEF domains. 3'dGTP/Mg^{++} as found bound to DgcR'* (cartoon) is shown with yellow carbons and light green spheres. 3'dGTP/Mg^{++} as found bound to DgcR' is shown with pink carbons and dark green spheres. The 3'-OH groups of 3'dGTP have been reconstructed. GTP-αS/Ca^{++} as bound to DosC (4ZVF) [http://doi.org/10.2210/pdb4ZVF/pdb] is shown with pink carbons and a grey sphere.

Supplementary Fig. 6

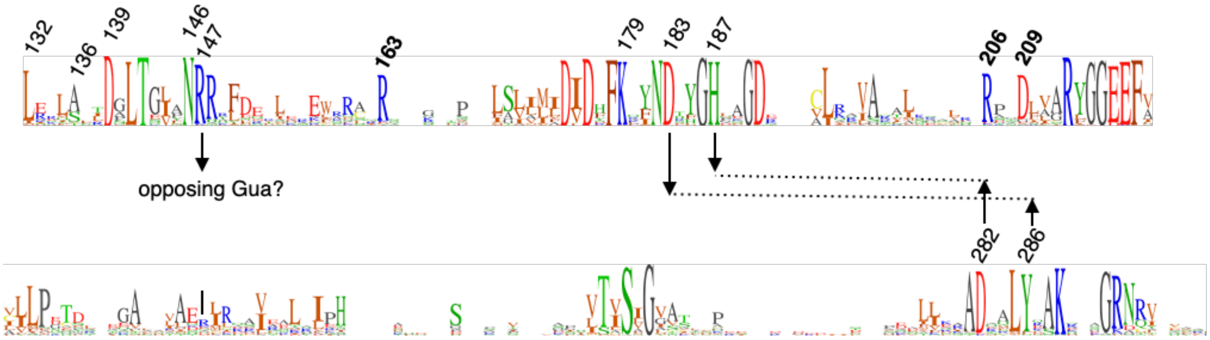

Supplementary Fig. 6. Sequence logo encompassing the GGDEF domain of Rec - GGDEF DGCs. The logo has been derived from the DgcR homologs of group 1 (see Fig. 10). Important residues are indicated by their number in DgcR (in bold for residues involved in c-di-GMP feed-back inhibition, see Fig. 8). Arrows indicate putative residues engaged in inter-domain contacts in the competent GGDEF dimer arrangement (Fig. 6b). R147 may interact with the Hogsteen-edge of the guanyl-base of the substrate bound to the opposing domain.

Supplementary Fig. 7

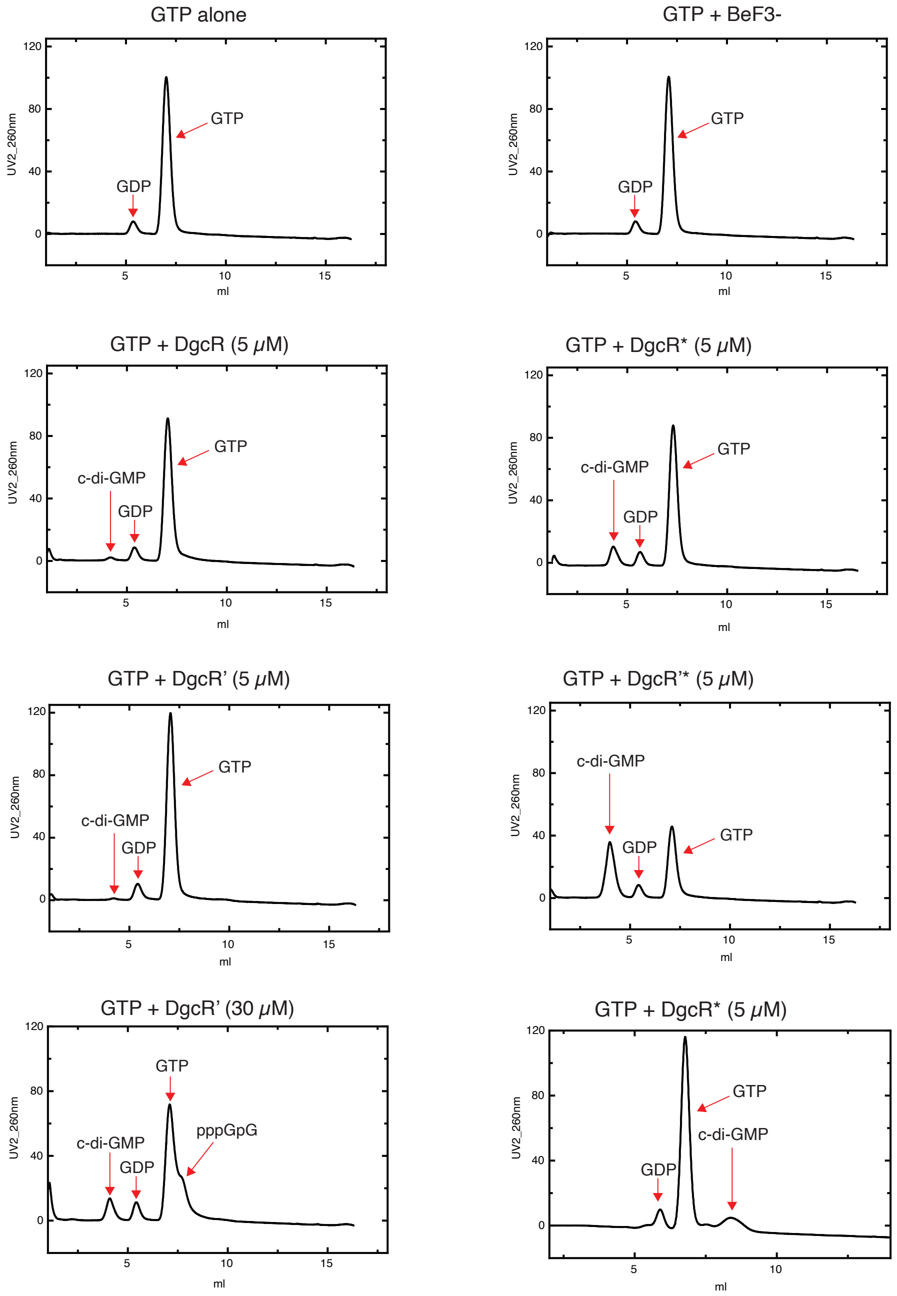

Supplementary Fig. 7. Elution profiles of DgcR reactions. Blank runs composed by **a)** GTP alone and **b)** GTP with BeF_3^- showing that BeF_3^- does not react with GTP. **c-g)** Elution profiles after 16 mins incubation of 500 μM GTP with 5 μM of the indicated DgcR variants eluted using 10 mM HCl with a 0 - 400 mM NaCl gradient. **h)** Same as **d),** but eluted with a 0 - 1 M ammonium sulfate gradient in absence of HCl (as used for the experiments of Fig. 9). Note that the reaction intermediate pppGp is detectable only when using DgcR' (unmodified by BeF_3^-) at high concentrations (30 μM).

Supplementary Tables

Supplementary Table 1. Data collection and refinement statistics.

	DgcR'	DgcR'*	DgcR_inh
**Protein**	DgcR_AxxA/3'dGTP	DgcR_AxxA/$BeF3^-$;3'dGTP	DgcR_wt/c-di-GMP
**Data collection**			
Synchrotron source	SLS, PXIII	SLS, PXI	SLS, PXIII
Resolution	41 - 2.2 (2.3 - 2.2)	41 - 2.8 (2.9 - 2.8)	48 - 3.3 (3.4 - 3.3)
Space group	C 2	$P\ 2_1\ 2_1\ 2$	$P\ 2_1$
a, b, c (Å)	137, 39, 146	133, 247, 41	123, 73, 126
α, β, γ (°)	90, 109, 90	90, 90, 90	90, 118, 90
Total reflections	191690 (18770)	205975 (19551)	111858 (9780)
Unique reflections	38087 (3727)	34912 (3320)	29577 (2768)
Multiplicity	5.0 (5.0)	5.9 (5.7)	3.8 (3.5)
Completeness (%)	98 (98)	94 (97)	99 (94)
Mean I/sigma(I)	10.7 (1.2)	8.6 (1.5)	6.7 (1.6)
R-merge	0.09 (1.20)	0.29 (1.13)	0.17 (0.75)
R-pim	0.05 (0.60)	0.13 (0.51)	0.10 (0.46)
CC1/2	0.99 (0.70)	0.90 (0.60)	0.99 (0.70)
**Refinement**			
R-work/R-free (%)	22.6/25.9	23.3/28.8	22.4/29.9
Number of molecules/a.u	2	4	6
Number of atoms	4804	9545	14628
protein	4672	9312	14070
ligands	101	184	558
RMS bonds (Å)	0.012	0.010	0.011
RMS angles (Å)	1.67	1.86	1.98
Ramachandran favored (%)	95.02	96.79	85.45
Ramachandran allowed (%)	4.12	2.78	11.17
Ramachandran outliers (%)	0.86	0.43	3.38
Average B-factor ($Å^2$)	66.65	71.67	96.76
protein	66.90	71.83	97.45
ligands	65.47	63.73	79.53
water	45.56	72.58	55.87
PDB code	6ZXB	6ZXC	6ZXM

Statistics for the highest-resolution shell are shown in parentheses.

Supplementary Table 2. Kinetic parameters of DgcR diguanylate cyclase activity

	K_d (µM) #	k_{cat} (s^{-1})	inhibition type	k_{off} (s^{-1})	K_i (µM)
**DgcR**	10	$(7.3 \pm 0.9) \cdot 10^{-3}$	non-competitive, equilibrium	n/a	26 ± 10
**DgcR***	10	0.33 ± 0.03 ##	non-competitive, slow	$(0.58 \pm 0.08) \cdot 10^{-3}$<br>$(1.9 \pm 0.3) \cdot 10^{-3}$ ##	0.11 ± 0.01<br>0.21 ± 0.03 ##
**DgcR'**	10	$(1.5 \pm 0.03) \cdot 10^{-3}$	none	n/a	n/a
**DgcR'***	10	0.33 ###	non-competitive, slow	$(1.3 \pm 0.4) \cdot 10^{-2}$	6.3 ± 0.2

Parameters and errors as derived from fitting of the kinetic model of Fig. 9d to the data in Fig. 9b.

set arbitrarily to reasonable value. Since it is much smaller than employed substrate concentration (500 µM), exact value does not affect calculations.

as measured by conventional IEC (Fig. 9a).

set equal to DgcR'* value.

Supplementary Table 3. Primers used for generating DgcR' variant (R206A/D209A)

Forward	5' GCC AAA GCG ACT GAT AAT AGC AGA TTT AGC GAA GCT GGT TTT CAG TTC G 3'
Reverse	5' CGA ACT GAA AAC CAG CTT CGC TAA ATC TGC TAT TAT CAG TCG CTT TGG C 3'
