# Supplementary material for: Activation mechanism of a small prototypic Rec-GGDEF diguanylate cyclase
Source: Nat Commun. 2021 Apr 12;12:2162. doi: 10.1038/s41467-021-22492-7 (PMC8041772; doi:10.1038/s41467-021-22492-7)
Supplement: Supplementary file 6 — Description of additional supplementary files [file 41467_2021_22492_MOESM6_ESM.docx]

Description of additional supplementary information

Title: Supplementary movie 1

Description: Structural transitions in DgcR upon Rec pseudo- phosphorylation (side view).

Title: Supplementary movie 2

Description: Top view of structural transitions in DgcR upon Rec pseudo-phosphorylation (top view).
